# Commencement of Preliminary Term at Buffalo Medical College

**Published:** 1853-10

**Authors:** 


*Commencement of Preliminary Term at Buffalo Medical College.*—The preliminary term commences on Wednesday, October 5th. It will be devoted to practical anatomy, and to clinical instruction at the Hospital of the Sisters of Charity. The prospects of the school are flattering, and it is expected that the attendance of students will be large. Efforts will be made on the part of the faculty, to render an attendance on the preliminary term, advantageous and pleasant to their pupils. S. B. H.

